# Exploring Optimal Strategies for Surgical Access in Transcatheter Aortic Valve Implantation

**DOI:** 10.3390/jcm13164655

**Published:** 2024-08-08

**Authors:** Rushmi Purmessur, Zeba Ahmed, Jason Ali

**Affiliations:** 1 Department of Cardiac Surgery, Royal Papworth Hospital NHS Foundation Trust, Cambridge CB2 0AY, UK; jason.ali@nhs.net; 2 Department of Histopathology, University Hospitals Birmingham NHS Foundation Trust, Birmingham B15 2GW, UK

**Keywords:** transcatheter aortic valve implantation, surgical access, transaxillary, transcarotid, transaortic, transapical

## Abstract

Transcatheter aortic valve implantation (TAVI) has revolutionised the management of severe aortic stenosis, particularly for patients deemed high risk or inoperable for traditional surgical aortic valve replacement. The transfemoral approach is the preferred route whenever feasible, attributed to its minimally invasive nature, reduced procedural morbidity, and shorter recovery times. In total, 80–90% of TAVI procedures are performed via the transfemoral route. However, anatomical constraints such as severe peripheral arterial disease, small vessel diameter, or significant vessel tortuosity can preclude the use of this access site. In such cases, alternative access strategies must be considered to ensure the successful implantation of the valve. This review aims to provide a comprehensive summary of the various surgical techniques available for TAVI access, exploring the rationale, technical aspects, and challenges associated with each method. We will explore alternative routes, including the transapical, transaortic, transaxillary, and transcarotid approaches, highlighting their respective benefits and limitations.

## 1. Introduction

Transcatheter aortic valve implantation (TAVI), also known as Transcatheter Aortic Valve Replacement (TAVR), has emerged as a revolutionary management strategy for patients with severe aortic stenosis who are deemed high risk or inoperable for traditional surgical aortic valve replacement (SAVR). TAVI offers a less invasive alternative to open-heart surgery, allowing for the replacement of the aortic valve through percutaneous techniques. As the population ages and the prevalence of aortic stenosis rises, the demand for TAVI procedures continues to grow.

The concept of transcatheter balloon-expandable valves was pioneered by Dr Henning-Rud Andersen in the 1980s [1]. The first transcatheter aortic valve replacement was performed in 2002 by Dr Alain Cribier and his team in France [2,3], ushering in a new era in the treatment of valvular heart disease. The development of TAVI represents a significant milestone in the field of interventional cardiology, transforming the management of aortic stenosis, particularly in elderly and high-risk patients. The initial iterations of TAVI procedures primarily utilised transfemoral venous access, whereby the catheter is passed transseptally through the interatrial septum into the left atrium, left ventricle, through the aortic valve, into the ascending aorta, arch of the aorta, descending aorta, and eventually threaded to the femoral artery before deployment of the transcatheter heart valve (THV) [3]. However, soon after, a retrograde delivery system was developed wherein the THV was delivered via the femoral artery [4]. This approach revolutionised the management landscape by offering a minimal access alternative to traditional surgical techniques.

The transfemoral approach remains the preferred route whenever feasible, owing to its less invasive nature, reduced procedural morbidity, and shorter recovery times. Indeed, in clinical practice, 80–90% of THV are delivered through a transfemoral route [5,6]. In the UK, according to the 2024 NICOR report of the TAVI registry, 96% of TAVI procedures are performed through a percutaneous transfemoral route [7]. However, anatomical constraints, such as iliofemoral artery disease, aortic arch pathology, vessel tortuosity, presence of previously implanted arterial grafts, or severe peripheral vascular disease, may preclude transfemoral access and necessitate alternative strategies (Table 1). Recently, intravascular lithotripsy (IVL) has emerged as a new technology that fractures intimal and medial calcifications, improving vessel compliance. This advancement allows for transfemoral TAVI in select patients with severe peripheral artery disease [8].

As a result, over time several alternative access routes have been increasingly employed, maintaining the minimal access nature of TAVI, but requiring the input of the surgical team to facilitate obtaining arterial access. Alternative access options commonly utilised in such patients include: transapical through the left ventricular apex, transaortic through the ascending aorta, and via central arteries including the carotid, subclavian, and axillary. For patients with unsuitable arterial access who are not candidates for transapical TAVI, Greenbaum et al. developed the transcaval TAVI technique. This approach involves accessing the femoral vein, crossing into the abdominal aorta with a wire, and proceeding retrogradely into the ascending aorta [9]. Despite its innovative nature, the transcaval technique has not gained widespread adoption due to the favourable safety profile of transfemoral arterial access.

**Figure 1:**
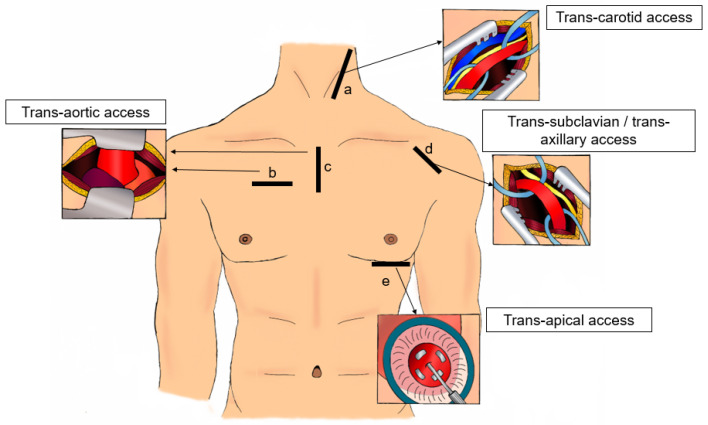
a. Trans-carotid access. Incision along anterior border of sternocleidomastoid muscle. b. Transaortic access. Right anterior mini-thoracotomy in 2nd or 3rd intercostal space. c. Mini upper partial sternotomy to 2nd or 3rd intercostal space. d. Trans-subclavian/transaxillary access. Incision in the delto-pectoral groove. e. Transapical access. Left anterior mini-thoracotomy in infra-mammary fold or 4th or 5th intercostal space.

Here, we will review in turn the different techniques currently available for obtaining surgical access for TAVI procedures, considering the rationale, technique, and challenges of each (Figure 1).

## 2. Anatomical Considerations and Pre-Procedural Imaging

Preprocedural imaging is vital for assessing vascular anatomy, identifying potential access sites, and predicting procedural success in TAVI. Successful outcomes hinge on careful planning and individualised approaches, established by a multidisciplinary team that combines the expertise of medical, radiological, and surgical professionals.

Computed tomography angiography (CTA) is the cornerstone investigation for evaluating vascular anatomy and determining the suitability of access points in TAVI [10,11]. Specifically, multidetector computed tomography (MDCT) offers high spatial resolution in three dimensions and allows for 3D reconstruction of images, providing a comprehensive evaluation of the vessel beyond the limitations of the transverse axial plane. Key characteristics to consider include vessel size, degree of calcification, minimal luminal diameter, plaque burden, vessel tortuosity, presence of dissections, aneurysms, [12] Dacron grafts for bypasses, and complex atheroma. MDCT is also crucial for annular sizing and coronary artery assessment [13,14].

For patients with significant renal impairment (eGFR < 30 mL/min), magnetic resonance angiography (MRA) without contrast serves as an alternative imaging modality. Intravascular ultrasound (IVUS) has also been explored, offering high-resolution, three-dimensional vessel examination without the risk of blooming artifacts around plaques and calcifications [13].

Vessel examination is paramount because the vessel must accommodate the sheath used for transcatheter heart valve (THV) deployment. The sheath fit should not be so snug as to occlude blood flow completely. Although sheath sizes have decreased since the initial use of THVs, with initial sheaths having an internal diameter of 8.4 mm and 9.2 mm [6], the internal diameters of sheaths currently range from 6.2 mm to 9.2 mm, with external diameters approximately 1 mm larger. In non-calcified arteries, which are more compliant, a sheath can be inserted through an artery approximately 0.75 times the sheath’s outer diameter, requiring a minimum vessel diameter of 5.5 mm. Conversely, in heavily calcified arteries, which are stiffer, a minimum vessel diameter of 1.25 times the sheath’s external diameter is advisable [12,14].

A certain amount of vessel tortuosity can be managed using stiffer wires, which can straighten out non-calcified arteries. However, in cases of very tortuous and heavily calcified vessels wires can snag and kink, hindering successful access and deployment of the THV and increasing the risk of vessel injury [12,14]. Therefore, thorough preprocedural imaging and meticulous planning are essential for optimising patient outcomes in TAVI. 

## 3. Transaxillary/Trans-Subclavian Access

**Figure 2:**
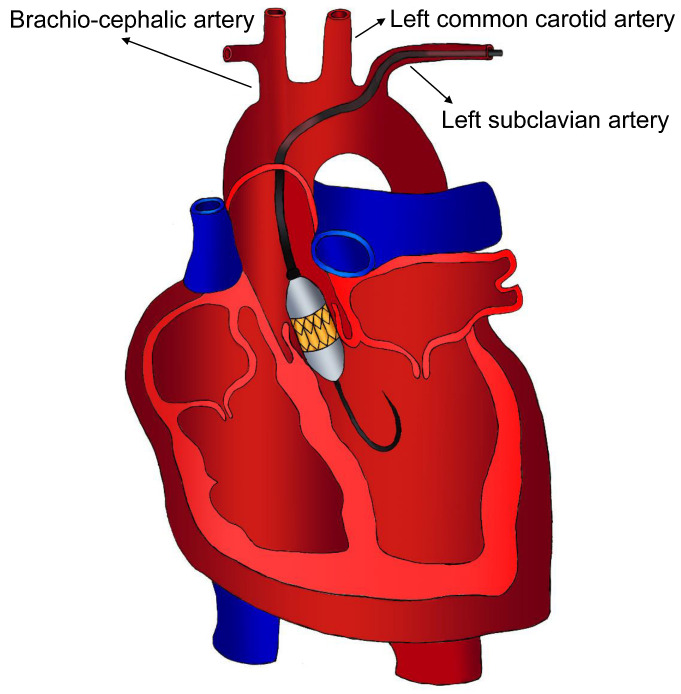
Trans-subclavian/subaxillary access.

One of the advantages of transaxillary or trans-subclavian access (Figure 2) is the proximity and relatively straight course from the axillary artery to the aortic annulus, facilitating more accurate device deployment.

### 3.1. Pre-Procedural Considerations

Critical points specific to the axillary or subclavian arteries to be evaluated are the relationship with side branches and the presence and extension of calcifications, particularly at the aortic take-off of the subclavian artery [11]. Special attention must be placed on identifying subclavian stenosis, which can compromise arterial patency and hinder sheath advancement, increasing the risk of access-related complications and procedural failure. Patients with significant subclavian stenosis may not be suitable candidates for transaxillary or trans-subclavian access.

### 3.2. Size of the Artery

The size of the axillary or subclavian artery plays a crucial role in determining the feasibility and safety of transaxillary or trans-subclavian access in TAVI procedures. In the absence of significant calcification, the arterial diameter should ideally be at least 6 mm to accommodate the passage of an 18 French (Fr) sheath [13], which is commonly used for THV delivery systems.

### 3.3. Left Axillary Artery vs. Right Axillary Artery

The left axillary artery is often preferred for TAVI procedures due to several inherent advantages. Firstly, accessing the left axillary artery allows for better coaxial orientation during THV deployment, facilitating optimal positioning and deployment accuracy. Secondly, utilising the left axillary artery decreases the risk of carotid artery compromise compared to right-sided approaches [15]. Given the shared arch origin of the right carotid artery and right subclavian artery, accessing the left axillary artery reduces the likelihood of inadvertent carotid artery injury or occlusion during sheath insertion and manipulation. Several anatomical factors influence the feasibility and safety of left axillary artery access in TAVI procedures. The aortic annular angle and the take-off angulation of the subclavian and innominate arteries with the aortic arch are critical determinants of access route suitability. An aortic annular angle >30° between the annular plane and the right subclavian horizontal axis, or >70° between the annular plane and the left subclavian horizontal axis (i.e., “horizontal aorta”) typically represents a contraindication to a right-sided approach [10,15]. These angulations pose challenges in achieving coaxial alignment during THV deployment, increasing the risk of mal-positioning and procedural complications. Furthermore, anatomical variations such as Type 1 aortic arch configuration (where all three great vessels originate directly from the transverse arch) may influence access route selection. Right-sided approaches may be avoided in Type 1 arches, particularly if the innominate artery arises distally on the arch, as this can complicate sheath navigation and deployment. Conversely, left-sided access may present challenges in cases where the left subclavian artery is retroflexed towards the descending aorta or exhibits a steep subclavian-to-arch angulation (>80°) [10]. These anatomical variations can hinder sheath advancement and positioning.

### 3.4. Surgical Technique

Conscious sedation with local anaesthesia or general anaesthesia can both be used. The patient is placed supine and draped, ensuring that the deltopectoral groove is exposed. A horizontal 5–6 cm incision is made about 2 cm below and at the junction of the middle and outer thirds of the left clavicle, overlying the deltopectoral groove. Using the diathermy as a spatula, the pectoralis muscle fibres are separated, and a self-retaining retractor inserted. The clavipectoral fascia is incised using Metzenbaum scissors, exposing the pectoralis minor muscle. This is divided and the self-retaining retractor is further advanced. There is usually a fat pad overlying the axillary artery, which is dissected out. The axillary artery usually lies superior and posterior to the vein and can be identified by palpation. Care must be taken not to disturb the medial and lateral cords of the brachial plexus by sharp dissection and avoiding unnecessary traction. A branch of the axillary artery is almost always just medial to the pectoralis muscle, which dips from the posterior aspect of the artery—care must be taken not to injure this branch. The axillary artery is then gently mobilised for about 2–3 cm and looped distally and proximally. At this point, 5000 IU of Heparin is given and a needle inserted into the artery to serve as an introducer for the guidewire. Given the angled course of the subclavian artery at the vertebral artery branch, a more kink-resistant sheath is used for delivery [13]. Once the valve has been deployed, the sheath is removed, protamine given, and the defect within the artery sutured. The wound is then closed in the standard fashion as used in each unit, ensuring thorough haemostasis [16].

### 3.5. Patient Outcomes

Studies report low rates of access-related complications and procedural success rates comparable to transfemoral approaches [17,18,19]. Petronio et al. (2012) [20,21] published their 2 year outcomes of the Medtronic-CoreValve (Medtronic, Minneapolis, MN, USA) implantation through the subclavian access. The study compared outcomes of TAVI via subclavian vs. femoral access. Procedural success rates were similar (97.9% subclavian vs. 96.5% femoral, *p* = 0.47), as were major vascular complications (5.0% vs. 7.8%, *p* = 0.33), life-threatening bleeding (7.8% vs. 5.7%, *p* = 0.48), and combined safety endpoint (19.9% vs. 25.5%, *p* = 0.26). Subclavian access showed lower rates of acute kidney injury (4.3% vs. 9.9%, *p* = 0.02) and minor vascular complications (2.1% vs. 11.3%, *p* = 0.003). Survival at 2 years and freedom from cardiovascular death were comparable between groups (*p* > 0.05).

Although transaxillary/trans-subclavian access is primarily surgical, it should be noted that Schafer et al. in 2012 [22] described a percutaneous subclavian TAVI approach called the “Hamburg Sankt Georg approach” and in 2017 [23] published their results on 100 patients. In their study, transaxillary TAVI, with a mean age of 78.2 years and 24.6% logEuroSCORE I, had 95% device success. Mortality rates at 30 days and one year were 6% and 14.8%, respectively.

## 4. Trans-Carotid Access

**Figure 3:**
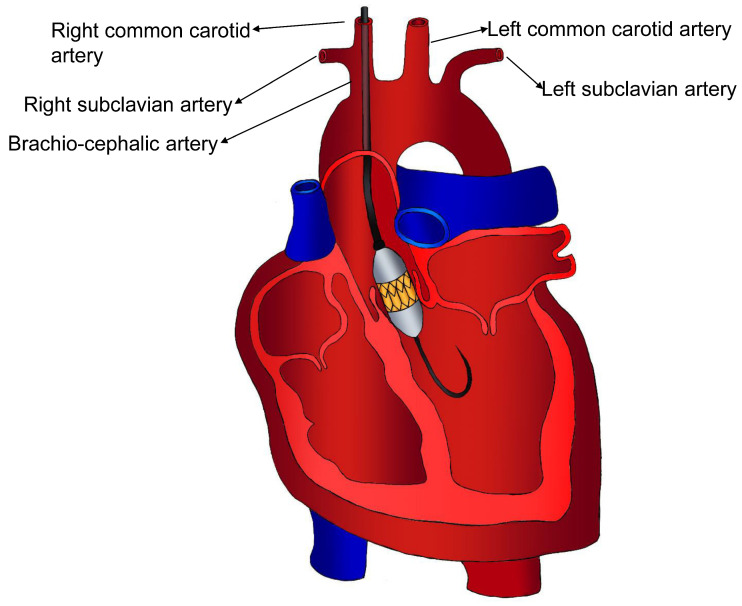
Trans-carotid access.

Trans-carotid access (Figure 3) offers several advantages in TAVI over a transfemoral or transaxillary/trans-subclavian approach. Its proximity to the aortic plane allows for controlled valve delivery, typically from the left side, enhancing procedural precision. Moreover, procedures can often be performed under local anaesthesia, minimising the risks associated with general anaesthesia and enabling faster patient recovery. This approach is particularly beneficial for patients who are poor candidates for a general anaesthetic but lack suitable transfemoral access. Importantly, trans-carotid access avoids crossing the diseased aortic arch and descending aorta, minimising the risk of debris embolisation associated with prolonged contact during aortic arch navigation, which is a potential complication of transfemoral and transaxillary/trans-subclavian access. In exceptional cases, such as patients with residual dissection of the distal aortic arch post-type A aortic dissection or associated type B dissection, trans-carotid access may be the only viable option due to anatomical considerations [24,25]. Left carotid artery surgical access may be necessary in cases of severe iliofemoral and subclavian artery tortuosity or stenosis, or to avoid transapical or transaortic access. Additionally, the presence of a pacemaker in the left subclavian area or prior coronary artery bypass grafting with a patent left internal thoracic artery may warrant trans-carotid access [24,25].

### 4.1. Pre-Procedural Considerations

Trans-carotid access for TAVI requires specific anatomical criteria and careful patient selection. The common carotid artery must have a diameter greater than 5.5–7.5 mm, depending on the institution, with patency of the circle of Willis confirmed via angiographic computed tomography or magnetic resonance angiography to determine the adequacy of cerebral collateral blood flow. Contraindications include common carotid artery diameter less than 5.5 mm, significant tortuosity, ipsilateral common carotid calcifications, occlusion of any vertebral artery, and high-risk atherosclerotic plaques. Previous carotid surgery on the ipsilateral side, untreated contralateral common carotid artery stenosis (≥50%), congenital variants of the aortic arch, or absence of patency in the circle of Willis also contraindicate trans-carotid access [26].

### 4.2. Surgical Technique

Trans-carotid access can be performed either under local or general anaesthetic. Intra-operatively, it is recommended to continually monitor cerebral perfusion using cerebral oximetry with near-infrared spectrometry. Systolic blood pressure should be maintained above 100 mmHg throughout the procedure. The patient is placed in supine position and draped, ensuring that the neck on the chosen side is exposed. The common carotid artery bifurcates approximately 2.5 cm below the angle of the mandible, typically bound by the sternocleidomastoid, digastric, and omohyoid muscles. A 5 cm incision is made along the anterior border of the sternocleidomastoid muscle, which exposes the carotid sheath. Careful dissection is essential to protect important nerves and vessels. The facial nerve’s mandibular ramus is at risk of injury, especially when the head is turned towards the opposite side. Protection of the great auricular nerve is vital to prevent distressing ipsilateral occipital headaches. Additionally, awareness of the common facial vein, hypoglossal nerve, and vagus nerve anatomy is essential to avoid inadvertent damage during surgery. Once exposed, the carotid artery is mobilised, and proximal and distal control is obtained using vessel loops. At this point, 5000 IU of Heparin is given and a needle inserted into the artery to serve as an introducer for the guidewire. Once the THV has been deployed, the sheath is removed, protamine given, and the defect within the artery sutured. The wound is then closed in standard fashion, ensuring thorough haemostasis [24,25,26,27,28,29,30].

### 4.3. Patient Outcomes

A meta-analysis by Lu et al. in 2021 [31] compared outcomes of trans-carotid and transfemoral TAVI in 1374 trans-carotid patients and 3706 transfemoral patients. Trans-carotid TAVI patients had higher EuroSCORE II and Logistic EuroSCORE values (respectively, 8.0 ± 6.7 vs. 6.3 ± 5.4, *p* = 0.002 and 20.8 ± 14.2% vs. 20.0 ± 13.4%, *p* = 0.04), more peripheral arterial disease (52.6 vs. 32.8%, *p* = 0.001), prior cardiac surgery (26.3 vs. 22.4%, *p* = 0.008), and coronary artery disease (64.6 vs. 60.5%, *p* = 0.020). Trans-carotid TAVI was associated with significantly higher 30 day mortality risk (RR, 1.41, 95% CI, 1.02–1.96, *p* = 0.040), but lower major vascular complications (RR, 0.48, 95% CI, 0.25–0.92, *p* = 0.030). There were no significant differences in permanent pacemaker implantation, major bleeding, or acute kidney injury. However, trans-carotid TAVI showed an increased risk of 30 day neurovascular complications (RR, 1.61, 95% CI, 1.02–2.55, *p* = 0.040). Abraham et al. in 2023 [32] published a meta-analysis comparing trans-carotid access to alternative routes, including 22 observational studies and a total of 11896 patients. The study assessed outcomes during hospitalisation and at 1 month follow-up. Trans-carotid TAVI exhibited higher 1 month mortality (3.7% vs. 2.6%, *p* = 0.02) compared to transfemoral access, albeit with fewer major vascular complications (1.5% vs. 3.4%, *p* = 0.04) during hospitalisation. It also demonstrated lower major vascular complications compared to transaxillary/subclavian access (2% vs. 2.3%, *p* = 0.04) but higher major bleeding rates (5.3% vs. 2.6%, *p* = 0.03). However, it did not elevate stroke risk compared to other access routes. 

## 5. Transaortic Access

**Figure 4:**
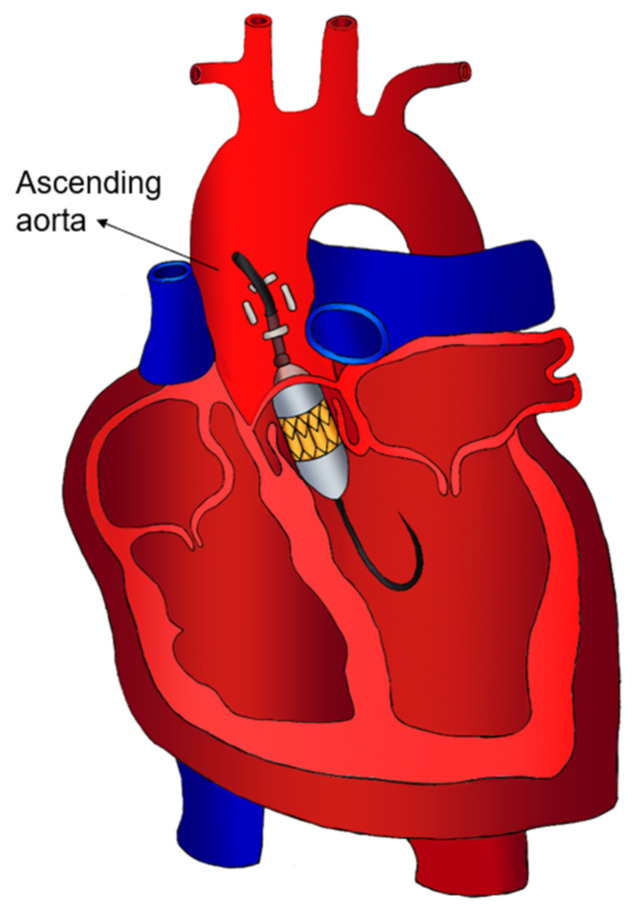
Transaortic access.

Transaortic TAVI (Figure 4) offers an alternative in patients with severely calcified peripheral vessels and, specifically, a diseased aortic arch. Transaortic TAVI confers several advantages. It allows for the utilisation of partial sternotomy or right anterior thoracotomy and ascending aorta cannulation, techniques commonly practiced in cardiac surgery, which enhance procedural familiarity and ease of access. Furthermore, when a partial upper sternotomy is employed, the transaortic approach allows for rapid conversion to a full sternotomy in the event of severe complications, providing the surgical team with enhanced control and manoeuvrability. Compared to the transapical approach, transaortic TAVI is associated with reduced risks of myocardial damage, apical bleeding, and impaired healing, potentially leading to improved postoperative outcomes. 

### 5.1. Pre-Procedural Considerations

Direct aortic access is particularly advantageous in cases of severely atherosclerotic aortic arches, minimising the risk of embolisation associated with extensive manipulation. Pre-procedural evaluation of aortic wall quality and trajectory analysis are essential for optimal alignment and valve release [11]. Meticulous assessment of the aortic wall quality where purse string sutures will be placed is essential, with a requirement for an area free of calcium spanning at least 1 cm^2^ [11,32]. This evaluation is typically conducted through CT scan analysis, often achievable with a contrast-free scan. Additionally, consideration of the trajectory between the entry site and the aortic valve annulus is performed to ensure optimal alignment between the delivery system and the native aortic valve. An ascending aorta with a horizontal orientation, characterised by an angle exceeding 70°, may necessitate increased manipulation of the delivery system, heightening the risk of valve misalignment. Furthermore, to facilitate the complete release of the valve, the distance between the aortic entry and the aortic annulus should be at least 6 cm [11]. The few contraindications to transaortic TAVI include thoracic deformities, short ascending aorta, porcelain aorta, and patent venous coronary artery bypass grafts at risk of damage.

### 5.2. Surgical Technique

This procedure is done under general anaesthesia. The patient is placed in a supine position and draped according to the preferred access. There are two main access routes: partial upper sternotomy to the second or third intercostal space, or a right anterior mini-thoracotomy in the second or third intercostal space, with an aim to expose the mid-distal ascending aorta for cannulation. Mini J sternotomy is preferred when the ascending aorta is located in the midline or towards the left side and is approximately 6 cm deep to the sternum. Conversely, a mini right thoracotomy is favoured when the ascending aorta is situated on the right side, typically indicated by approximately 50% of the aorta’s presence to the right of the sternal border [33,34,35,36]. This approach is typically performed at the level of the second intercostal space and requires a depth of approximately 6 cm to access the ascending aorta effectively. In mini J sternotomy, the procedure typically begins with a skin incision made in the midline, extending from the top of the sternum to the level of the third rib. The sternum is then divided using an oscillating sternal saw, with a mini oscillating saw utilised to extend the sternotomy into the third intercostal space. A mini-sternal retractor is employed to open the sternum. The pericardium is opened longitudinally and the edges of the pericardium are secured to the retractor, thereby bringing the aorta into closer view and allowing continuous lung ventilation without obstructing the operative field. Conversely, in mini right thoracotomy, the approach involves entering through the second intercostal space. A 4 cm skin incision is made along the right sternal border, followed by entry into the pleural space. The right internal mammary vessels may be retracted or ligated to optimise exposure [33]. The ascending aorta is then accessed using either a soft-tissue retractor alone or in combination with a small rib spreader. Thymic tissue is dissected, and the pericardium is identified and incised parallel to the incision, with pericardial stay sutures used to retract it and expose the ascending aorta. Once the site for purse strings is identified, two circular purse strings are placed using Polypropylene 3-0 sutures. The first purse string is non-pledgeted, while the second is pledgeted with four Teflon pledgets, sized appropriately for accommodating the sheath used for the TAVI device. Finally, the sutures are secured with snares, preparing the site for subsequent valve implantation. After systemic heparinisation to achieve an activated clotting time >300 s, a needle is inserted in the centre of the purse strings and the implantation of the valve performed in a standard fashion. The pericardium is left open, and a drain inserted into the pericardial space for a mini J sternotomy or into the right pleural space for a right anterior mini thoracotomy. One needs to be aware of the complications of a transaortic approach, which include ventricular perforation/injury owing to an extra stiff guidewire or intravenous pacing lead and aortic dissection [33,34,35,36,37,38,39].

### 5.3. Patient Outcomes

There are no major randomised controlled trials specifically comparing the outcomes of transaortic and alternative routes of access for TAVI [37,39,40]. In an analysis comparing outcomes of propensity score matched patients undergoing transaortic vs. transfemoral TAVI by O’Hair et al. [41] in 2018, at 30 days the all-cause mortality rate was significantly higher in the transaortic group compared to the transfemoral group (10.9% vs. 4.1%, *p* < 0.001). This was corroborated by another meta-analysis by Abraham et al. [31], which compared the transaortic to the trans-carotid approach (in-hospital mortality, 11.7% vs. 1.9%, *p* = 0.02; 1 month mortality, 14.4% vs. 3.9%, *p* = 0.007). However, this disparity decreased at 1 year (28.1% vs. 23.2%, *p* = 0.063). Similarly, the combined endpoint of all-cause mortality or major stroke was notably elevated for the transaortic group both at 30 days (13.5% vs. 5.3%, *p* < 0.001) and at 1 year (30.4% vs. 24.2%, *p* = 0.025). Additionally, major/life-threatening bleeding and acute kidney injury were significantly more frequent in the transaortic group at 30 days (66.7% vs. 35.4% and 19.7% vs. 10.0%, respectively, both *p* < 0.001). The ROUTE registry reported a valve success rate of 96.7%. At 30 days, the mortality rate was 6.1%, with procedure-related mortality accounting for 3.1% of cases. Paravalvular regurgitation of moderate or severe classification occurred in 3.3% of patients. Permanent pacemaker implantation was required in 8.8% of cases [42].

## 6. Transapical Access

**Figure 5:**
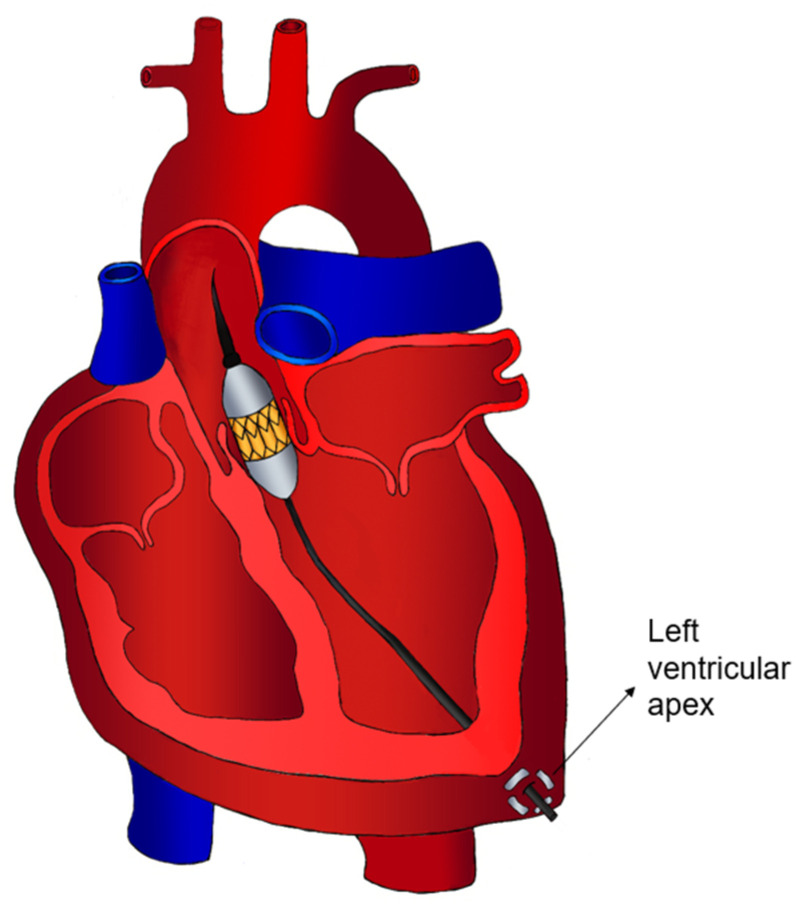
Transapical access.

Introduced in 2005, the transapical approach (Figure 5) offers a valuable alternative for TAVI in cases of unfavourable peripheral vascular anatomy. It allows for bypassing the compromised vascular tree entirely, providing excellent control over catheter and prosthesis placement due to the short distance between access and target [43]. Despite its historical prominence, the transapical approach now serves as the third choice access route after transfemoral and transaxillary approaches in many centres. This shift can be attributed to two primary factors: technological advancements and clinical outcomes [43]. With advancements in technology, there has been a reduction in the incidence of peripheral vascular complications associated with transfemoral access. Catheter size has decreased and flexibility has improved, enabling the treatment of patients with smaller vessel calibres. According to the 2024 NICOR data, the incidence of transapical TAVI procedures has significantly declined from 13.56% in 2013/2014 to 0.43% in 2022/2023 [7]. This trend underscores the fact that transapical TAVI has become increasingly rare, now reserved primarily for patients for whom no other vascular access options are viable. While transfemoral access can now accommodate more than 90% of TAVI patients, extreme cases with smaller vessel calibres or severe calcifications pose challenges and may necessitate alternative approaches.

### 6.1. Pre-Procedural Considerations

It is worth noting that most patients undergoing transapical TAVI have hypertrophic ventricles, which are thick and resistant. However, in cases of dilated and dysfunctional ventricles, such as those seen in low-flow aortic stenosis or mitral insufficiency, the ventricular wall may be thinned and more fragile. In such cases, transapical access poses a greater risk and should be minimised whenever possible. Transapical TAVI is relatively contraindicated in cases of impaired left ventricular function and in the presence of significant parenchymal lung disease [6].

### 6.2. Surgical Technique

In transapical TAVI, the procedural approach mirrors conventional surgery, with patients typically undergoing general anaesthesia with endotracheal intubation. The patient is placed in a supine position and draped to expose the left anterior chest wall. Some operators may find it easier to insert a roll underneath the left hemithorax to slightly elevate the left side and spread the left ribs for better access [44]. The procedure involves making a left anterior mini thoracotomy, usually at the level of the cardiac apex in the fourth or fifth intercostal space, which may entail opening the pleural cavity. It should be noted that this can pose challenges, particularly in elderly or frail patients with respiratory impairments. Unlike conventional surgery, transapical TAVI avoids the need for extracorporeal circulation, aortic clamping, and cardioplegia. Once the cardiac apex is exposed, pledgeted sutures are inserted around the left ventricle apex as a purse string. The left ventricle is punctured under direct vision and the procedure carried out under fluoroscopic guidance. The selected prosthesis, determined based on pre-operative evaluations, is then inserted through a dedicated catheter and implanted with the heart beating, allowing for real-time assessment of the result. Following valve implantation, the catheter is removed, and the breach in the heart is oversewn. Closure is in standard fashion as used by the institution, being sure to insert a pericardial and pleural drain [6,43,44].

### 6.3. Patient Outcomes

Observational studies [45,46,47,48] and registries [49] have historically reported higher mortality rates with the transapical approach compared to transfemoral access. This difference is partly attributed to the typically higher pre-operative risk profile of transapical patients. Even after adjusting for these differences, transapical access remains associated with increased mortality and morbidity, including prolonged hospitalisation, bleeding, and reduced recovery of left ventricular function. Conversely, alternative percutaneous approaches, such as transaxillary [19] access, have shown comparable outcomes to transfemoral [50] access, making them a preferred second choice when femoral access is not feasible.

## 7. Conclusions

The choice of surgical access for TAVI represents a critical decision in patient management, necessitating a nuanced understanding of individual patient anatomy, comorbidities, and procedural requirements. While the transfemoral approach has become the preferred method due to its minimally invasive nature and favourable outcomes in most cases, alternative access routes such as transaxillary/trans-subclavian, trans-carotid, transaortic, and transapical approaches offer valuable options for patients with anatomical constraints or contraindications to femoral access (Table 2).

In 2017, the American College of Cardiology published an expert consensus paper led by Otto et al. [51] advocating for transfemoral TAVI as the first-choice approach whenever feasible. The authors emphasise that advancements in reducing sheath diameters have made transfemoral access viable for most patients, including those with lower body surface area. The paper also recommends considering transaxillary/trans-subclavian, transaortic, and transapical as second-line alternatives. In the UK, the 2024 NICOR report on the TAVI registry revealed that the second most common access, after transfemoral, was trans-axillary/trans-subclavian (1.7%) [7]. Trans-carotid and trans-caval (not described in this review) approaches are suggested as third-line options, contingent on the experience and volume of the operators and centres performing the procedures [51].

A meta-analysis, by Chandrasekhar et al. in 2015, [52] of 28 studies involving 17,020 patients undergoing TAVI between 2007 and 2013 compared outcomes between transfemoral and non-transfemoral approaches. Transfemoral access showed lower 30 day (4.7% vs. 8.1%) and 1 year mortality (16.4% vs. 24.8%), with higher vascular complications but lower surgical conversion rates. Bleeding and cerebrovascular event rates were similar between groups.

As outlined above, several studies have highlighted the importance of careful patient selection and procedural planning to optimise outcomes and minimise complications. The transaxillary/trans-subclavian and trans-carotid approaches have emerged as promising alternatives, demonstrating comparable outcomes to transfemoral access and superior outcomes to transapical and transaortic approaches in certain patient populations. Additionally, advancements in surgical techniques and technology continue to enhance the safety and efficacy of alternative access routes, expanding the therapeutic options available for patients undergoing TAVI. Ultimately, a multidisciplinary approach involving collaboration between interventional cardiologists, cardiac surgeons, and imaging specialists is essential to tailor the choice of surgical access to individual patient needs, ensuring optimal outcomes and improving patient care in the evolving landscape of TAVI.

## Figures and Tables

**Table 1:** Situations wherein the femoral artery is unsuitable for TAVI access.

Severe circumferential calcification
Severe tortuosity of the femoral artery
Femoral aneurysm
Insufficient diameter
Severe iliofemoral disease
Chronic type B dissections
Presence of implanted arterial grafts, e.g., in peripheral bypasses from the femoral artery—femoral–popliteal or femoral–femoral bypass grafts, presence of Dacron grafts attached to the femoral artery (relative contraindication)

**Table 2:** Summarising the different approaches for transfemoral and surgical access routes for TAVI.

Access Route	Approach	General Anaesthesia	Size of Vessel/mm	Advantages	Contraindications/Disadvantages
Transfemoral	Percutaneous (or surgical cutdown sometimes)	No	5.5–6.5	Percutaneous, less invasive, low peri-procedural morbidity	Small tortuous vessel, femoral aneurysm, severe circumferential calcification, chronic type B dissections, presence of bypass grafts from the femoral artery
Transaxillary/trans-subclavian	Surgical (or percutaneous possible)	Yes	>6	Straight course from artery to aortic annulus, easy surgical access, does not cross diseased descending aorta	Subclavian stenosis, presence of LIMA to LAD graft if left access is considered, occlusion of right common carotid at shared origin if right access is considered, chronic arch dissections, arch aneurysms
Trans-carotid	Surgical	No	>5.5–7.5	Proximity to aortic plane, avoids crossing diseased arch or descending aorta, can be done under local anaesthetic	Absence of patent circle of Willis, common carotid artery <5.5 mm, significant tortuosity, ipsilateral common carotid calcifications, occlusion of any vertebral artery, presence of high-risk atherosclerotic plaques, previous carotid surgery on ipsilateral side, untreated contralateral common carotid artery stenosis (≥50%), congenital variants of the aortic arch
Transaortic	Surgical	Yes	N/A	Alternative in patients with severe PVD or calcified aortic arch	Porcelain aorta, chronic ascending aorta dissection, extensive calcifications in the ascending aorta, thoracic deformities, short ascending aorta, patent venous coronary artery bypass grafts at risk of damage
Transapical	Surgical	Yes	N/A	Bypasses vascular tree completely, avoids diseased aorta (chronic dissection, porcelain aorta, etc.)	Left ventricular aneurysm, impaired left ventricular function, presence of significant parenchymal lung disease

N/A: not applicable; PVD: peripheral vascular disease.
